# A New Treatment for Dysentery

**Published:** 1894-04

**Authors:** 


A NEW TREATMENT FOR DYSENTERY.—In the Texas Sanitarium, Dr. J. W. Mixon has been using a Mexican domestic remedy, *chopparro amargoso,* in his practice, and on himself. He reports the following results:

1. In November, 1891, I had a protracted case of dysentery, complicated with an old fistula, which was operated on, and did nicely, and soon healed, but the tenesmus and dysenteric stools continued. Commenced to drink a decoction of *chopparro amargoso* as an experiment, and continued it three or four times a day for three weeks, when I was entirely free from dysentery, and able to ride horseback continuously.

2. Lately I had an attack of acute dysentery (for the first time since January, 1892); tenesmus, frequent stools, principally mucus mixed with blood.

*Treatment.*—Reduced daily fare one-half, and took ʒj. fl. ext. *amargoso* (or equivalent of decoction) three times a day. On the second day had the last stool of mucus tinged with blood; no more stools until the fourth day, which was normal Discontinued the *amargoso* on the fifth day; no symptoms up to now, two weeks since.

3. W. T. Magee, aged fifty years, contracted acute dysentery in May, 1890; stools rather large; at first fecal matter and mucus tinged with blood, and frequent tenesmus, also fever. Was treated with aperients, small doses of calomel, oil, seidlitz powders, etc., followed by opium, astringents, bismuth, etc. In a month was able to ride out on horseback. From over-exercise in riding he relapsed, and was treated again, but ineffectually. The case became chronic; went through all known medical treatment as suggested by many different physicians; traveled for a change of climate, etc., with only temporary relief, gradually growing weaker and emaciated. In January, 1892, he commenced drinking a decoction of *chopparro amargoso* four or five times a day. He improved from the first; tenesmus less severe, stools nearer normal. In one month patient was almost entirely relieved. Has had only slight symptoms occasionally, which were at once relieved by a few draughts of amargoso.

4. R. T. Knox, M. D., of Gonzales, about sixty years old, general practitioner, is an enthusiast on the subject, from the remarkable cure on himself by this drug, after exhausting the pharmacopœia. Hearing of this Mexican remedy, he tried *chopparro amargoso.* The doctor writes me: "I had chronic dysentery for three years (May, 1889, to May, 1892). I used every known remedy for relief, but they only held the bowels in check for a short while. I tried this remedy, and was much benefited. I will report my case in full soon."

He had grown so weak and emaciated that he had œdema of lower extremities, but now can be classed as a well man, notwithstanding the ravages produced by three years' dysentery.—*Ex.*

